# [Expression of Concern] CDKN2A (p16^INK4A^) affects the anti-tumor effect of CDK inhibitor in somatotroph adenomas

**DOI:** 10.3892/ijmm.2025.5680

**Published:** 2025-10-29

**Authors:** Yiyuan Chen, Zhenye Li, Qiuyue Fang, Hongyun Wang, Chuzhong Li, Hua Gao, Yazhuo Zhang

Int J Mol Med 47: 500-510, 2011; DOI: 10.3892/ijmm.2020.4807

Following the publication of the above paper, it was drawn to the Editor's attention by an interested reader that, regarding the western blot data shown in Fig. 5 on p. 507, the first set of GAPDH bands for the GH3 cell line were strikingly similar to the EGFR protein bands shown for the GT1-1 cell line in the adjacent set of gels, such that the same data had apparently been used to represent the two different proteins.

The authors were contacted by the Editorial Office to offer an explanation for the apparent duplication of data in this paper, although up to this time, no response from them has been forthcoming. Owing to the fact that the Editorial Office has been made aware of potential issues surrounding the scientific integrity of this paper, we are issuing an Expression of Concern to notify readers of this potential problem while the Editorial Office continues to investigate this matter further.

